# A cluster level study for the identification of the disparities in birth intervals between rural and urban areas of Bangladesh

**DOI:** 10.1371/journal.pone.0342304

**Published:** 2026-02-05

**Authors:** Farzana Afroz, Fatima Tuz Zahura, Shahnaz Nilima, Sabina Akter

**Affiliations:** 1 Department of Statistics, University of Dhaka, Dhaka, Bangladesh; 2 BRAC James P Grant School of Public Health, BRAC University, Dhaka, Bangladesh; Jahangirnagar University, BANGLADESH

## Abstract

Like most developing countries, it is pivotal to identify factors associated with birth intervals in Bangladesh for intervening programs to reduce maternal and under-five children deaths. In this study, an attempt has been made to examine the socioeconomic and demographic factors that influence birth intervals in rural and urban Bangladesh. For this purpose, the secondary data extracted from Bangladesh Demographic and Health Survey (BDHS), 2017−18 and 2022 have been utilized where a two-stage stratified sampling technique is used for data collection. The sample has been considered based on the information available on the birth interval for the last child of mothers. We have applied the Product-Limit approach, Log-rank test and the popular semi parametric frailty regression model that takes into account the possible correlation among observations from the same cluster. Slight disparity has been observed in the median birth intervals between urban (64 months, 2017−18; 67 months, 2022) and rural mothers (60 months, 2017−18; 63 months, 2022). In both datasets, age at marriage, women’s decision making autonomy, and division have been emerged as significant determinants of the birth intervals in rural and urban Bangladesh. In the 2017−18 data, the effect of partner’s education is limited to urban areas, while religion shows significance only in rural areas. Meanwhile, in the 2022 data, the wealth index and spousal age difference become significant only in rural areas. It is recommended that the government invest in programs to enhance women’s autonomy, since women’s greater autonomy significantly delays subsequent births in both rural and urban areas. According to the most recent 2022 data, rural women from middle class and rich families have significantly longer birth intervals compared to the poor families, hence promotion of family planning counseling should be more strongly promoted to them. In addition, analysis using 2022 data reveals that in rural areas, birth intervals initially shorten as the spousal age difference increases, then subsequently lengthen after a certain threshold; therefore, family planning initiatives in this area should incorporate guidance on selecting a proper age gap at marriage, which might contribute to longer birth intervals.

## Introduction

Fertility is a major component of population dynamics which determines the structure of a country’s population [1]. Birth interval, defined as the length of time between two successive live births, is a key indicator of fertility as well as socioeconomic development of a country [1]. The World Health Organization (WHO) recommends the interval of at least 33 months between two successive live births or 24 months for birth to conception of a pregnancy for better maternal and child health outcomes [2,3]. The interval shorter than WHO recommendation, defined as short birth interval (SBI) which is responsible for various maternal health complications such as malnutrition, anemia, toxemia, self-induced abortion and uterine rupture, and maternal mortality as well [2,4]. Globally, about 25% of live births occur with SBI, and a higher proportion of these births is observed in Central Asia and Sub-Saharan Africa [5,6]. SBI is strongly linked to adverse child health outcomes including preterm births, low birth weights (LBW) and small for gestational age [5–8], and it also influences babies’ standard physical and intellectual development [9,10]. Moreover, neonates and infants born in short birth intervals have a higher risk of dying [7,11]. Conversely, long birth intervals (more than 60 months) is also responsible for negative maternal and neonatal health outcomes such as induction of labor, chorioamnionitis, eclampsia, third trimester bleeding, Cesarean delivery, postpartum hemorrhage, premature birth, LBW and small for gestational age [4,12]. A study conducted in six low and lower middle-income countries demonstrates that women with long birth interval (LBI) had higher risk of maternal deaths, neonatal deaths, stillbirths, LBW, as compared to those with birth interval of 18–36 months [12]. Every year approximately 2·0 million stillbirths occur worldwide, and low-income and lower-middle income countries account for 83.6% of these stillbirths [13]. Optimal birth interval can avert most of the stillbirths, and hence results in reduced perinatal mortality rate [2]. Again, maternal nutrient depletion during pregnancy and lactation can be ameliorated by optimizing birth interval [2,4]. Therefore, optimal birth spacing is important for improvement of maternal and child health outcomes [2,6].

Although the median birth interval in Bangladesh has increased from 55.7 months in 2017−18 to 59.2 months in 2022, 10% of births still occur with SBI [14]. According to Bangladesh demographic and health survey 2017−18, neonatal, infant and under-five mortality rates were higher among children born with SBI compared to those for which the birth interval is found more than 2 years [15]. Similar scenarios have been observed in 2022 as well [14].

However, the third sustainable development goal target 3.2 aims to reduce neonatal and under-five mortality to less than 12 and 25 per 1000 live births, respectively which can be achieved through implementation of optimum birth interval. Furthermore, optimizing birth intervals is also required in order to meet SDG 3.1 target of decreasing maternal mortality ratio to at least 70 per 100000 live births.

Birth intervals can contribute to reducing a country’s population by controlling fertility [1,2]. In Bangladesh, the total fertility rate (TFR) has declined over the time and remained stable at 2.3 children per woman since 2011 [14]. Despite this progress, the Health, Population and Nutrition Sector Program (HPNSP) target of reducing TFR to 2.0 births per woman is still unmet. As birth interval helps to control population growth, improve maternal and child health outcomes; it is important for researchers and policy makers to identify the factors affecting birth interval in Bangladesh. A recent study conducted on short birth intervals has introduced women’s 3E jointly from women’s empowerment, education and economic status and found that the odds of experiencing SBI decreases as the coverage of 3E increases among women in Bangladesh [2]. This research also reveals that mother’s age at marriage, mother’s employment status, religion, survival status of the last child, and region are significant determinants of SBI. Khan et al. [1] analyzed pooled data derived from three Bangladesh Demographic and Health Surveys (BDHS) over the period 2004−2011 and observed that mother’s age at first birth, partner’s education, survival status of the child, wealth quintile, place of residence and division significantly affect birth interval [1]. Another research work performed by Mahmood and Zainab has demonstrated that maternal education, maternal age at birth and place of residence have potential impact on birth interval in Bangladesh [16]. However, Wegbom et al. conducted a study in Nigeria in order to observe the disparities among women residing in urban and rural Nigeria in terms of birth interval using logistic regression model [6]. The findings of their research suggests that mother’s age, region, mother’s education have significant association with SBI in both rural and urban Nigeria. According to this study, some disparities have been observed among urban and rural Nigerian women. To our knowledge, no research is conducted in Bangladesh to address the disparities in the factors influencing the birth interval among rural and urban women. The BDHS, 2017−18 report reveals urban-rural differences in birth spacing, with urban women exhibiting longer median birth interval (58.1 months) than rural women(54.5 months)[15]. This pattern also remains evident in BDHS 2022, indicating extended median birth interval among urban women compared with their rural counterparts (62.8 months versus 57.9 months) [14]. Moreover, rural women have consistently experienced higher TFR (2.3 births per women in 2017–18 and 2.4 births per women in 2022) than urban women (2.0 births per women and 2.1 births per women, respectively) [14,15]. Furthermore, both survey findings confirm that early neonatal deaths and stillbirths occur more frequently in rural areas than urban areas [14,15]. Since birth interval is strongly linked to fertility control and child mortality, it is important to investigate the disparities among the factors determining birth interval in urban and rural Bangladesh. Therefore, this study aims to identify the factors affecting birth interval in urban and rural areas of Bangladesh utilizing data sets obtained from BDHS, 2017−18 and 2022. Since both surveys used two-stage stratified cluster sampling, this study has performed analysis using a Frailty model in order to consider the unobserved correlation of birth intervals among mothers within the same cluster.

## Materials and methods

### Data source

The study utilized the data set on birth interval extracted from the nationally representative Bangladesh Demographic and Health Survey (BDHS), 2017−18 and 2022 data. Both the surveys employed a two-stage stratified sample of households for data collection. In the first stage, the BDHS 2017−18 survey sampled 675 clusters (enumeration areas (EA)) from 22 strata with probability proportional to EA size and the second stage consists of selecting 30 households from each EA chosen in the first stage. Finally, BDHS successfully interviewed 20,127 ever-married women of ages 15−49 years and collected information on their complete history of live births with measurements on some important background characteristics. For the BDHS 2022 survey, 237 urban and 438 rural EAs were selected in the first stage using probability proportional to EA size. In the second stage, a systematic sample of 45 households per EA was drawn to ensure reliable estimates for all eight divisions of Bangladesh. Ultimately, the survey successfully interviewed 30,078 eligible women of reproductive age. The sample design is discussed in detail in the final reports of BDHS, 2017−18 and 2022 surveys [15]. To get information for this study, the last births of mothers preceding five years of each survey have been considered which results in a sample size of 7384 (BDHS 2017–18) and 7,431 (BDHS 2022). However, two separate data sets for the analysis of birth intervals have been created for urban and rural areas in Bangladesh. The urban and rural sample sizes were 2,623 and 4,761, respectively, for BDHS 2017−18, and 2,505 and 4,926, respectively, for BDHS 2022.

### Ethics approval and consent to participate

The collection of BDHS, 2017−18 and 2022 were approved by the Institutional Review Board of ICF International, Rockville, MD, USA and Bangladesh Medical Research Council, Dhaka, Bangladesh. The survey was implemented by the authority of the National Institute of Population Research and Training (NIPORT) of the Government of the People’s Republic of Bangladesh under the financial support from USAID/Bangladesh. Prior to questioning, informed consent had been obtained from the respondents. Those who did not consent had not been interviewed.

### Study variables

The study focuses on disparities in birth intervals between urban and rural areas of Bangladesh. The outcome variable, preceding birth interval, is measured in months. The independent variables included in the analysis are: Mother’s Age at Marriage (MAM), Mother’s Education (ME), Media Exposure (MEX), Women’s Autonomy (WA), Exposure to Violence (EV), Religion (REL), Region (REG), Wealth Index (WI), Partner’s Education (PE), and Spousal Age Difference (SAD, measured in years). The definitions and measurement details of all variables are presented in S1 Table.

### Statistical methods

The study investigates the potential factors associated with birth intervals for urban and rural areas in Bangladesh. Kaplan–Meier survival curves are plotted to compare the survival probabilities across different categories of covariates, where the survival probabilities are estimated using the Product-Limit approach [17]. According to this method, if $n_{j}$ denotes the number of individuals at risk just before time $t_{j}$, and $d_{j}$ denotes the number of events occurring at $t_{j}$, then the survival probability at time t is calculated as follows:


$$\begin{array}{c} \hat{S}(t)=\prod {\mathrm{\backslash nolimits}}_{t_{j}\leq t}\left(\text{1}-\frac{d_{j}}{n_{j}}\right). \end{array}$$

Moreover, the log-rank test [18] has been applied to examine the unadjusted association between birth interval and the selected socio-economic and demographic variables. This method evaluates whether there is statistically any difference in the survival experience among different groups over time. The test statistic is calculated by comparing the observed and expected number of events at each time point, and it approximately follows a chi-squared distribution.

Since the data utilized in this study is a clustered data where enumeration areas serve as clusters, models that can incorporate random effects of clusters to explain the underlying heterogeneity should be an appropriate choice to obtain more precise results. Therefore, in this study, semi parametric frailty model [19] has been applied to incorporate random effects in the model in order to account for unobserved heterogeneity. The estimation technique of the model embarks penalized partial likelihood approach [20].

Assume that the data for subject *i*, who is a member under j^th^ of q families, follows proportional hazard shared frailty model. Also let X denote the observed covariate matrix of dimension n×p with β being the vector of corresponding regression parameters. The penalized regression formula under shared frailty model has been developed as [19],


$$\lambda_{i}(t)=\lambda_{\text{0}}(t)exp\;\left(X_{i}\beta +Z_{i}\omega \right),$$

where $\lambda_{i}(t)$ and $\lambda_{\text{0}}(t)$ are the hazard functions at time point t in the presence and absence of covariates, respectively, $\varpi_{j}=exp(\omega_{j})$ is the frailty term for j^th^ family which is assumed to be independent and identically distributed gamma variate with mean 1 and variance $\theta =\frac{\text{1}}{v}$ with $\omega_{j}$ being the random effect of j^th^ cluster, and Z is a n×q matrix with q indicator variables such that $Z_{ij}=\text{1}$, if subject *i* belongs to family *j,* and 0, otherwise. Under this set up, the penalized partial log-likelihood (PPL) function with g(ω;θ) being the penalty function for tuning constant θ can be written as [20].


PPL=PL(β,ω;data)−g(ω;θ);

where,

$Y_{i}(t)$ with $Y_{i}(t)$ being the indicator that a given subject is at risk and under observation at time point *t* and $N_{i}(t)$ is the cumulative number of events for the subject up to time t; and $g(\omega ;\theta )=-\frac{\text{1}}{\theta }\sum_{j=\text{1}}^{q}\left[\omega_{j}-exp(\omega_{j})\right]$.

In terms of the selected covariates, the frailty model employed in this study can be written as


$$\lambda_{i}(t)=\lambda_{\text{0}}(t)exp\;\left(\beta_{\text{1}}MAM+\beta_{\text{2}}ME+\beta_{\text{3}}MEX+\beta_{\text{4}}WA+\beta_{\text{5}}EV+\beta_{\text{6}}REL+\beta_{\text{7}}REG+\beta_{\text{8}}WI+\beta_{\text{9}}PE+\beta_{\text{1}\text{0}}SAD+\omega_{j}\right).$$

In the above model, the hazard of experiencing a birth outcome for *i*^*th*^ individual under *j*^*th*^ cluster is denoted by $\lambda_{i}(t)$, $\beta_{\text{1}},\;\beta_{\text{2}},\;\ldots ,\;\beta_{\text{1}\text{0}}$ are the regression coefficients for the corresponding covariates and the random effect of *j*^*th*^ cluster is incorporated as $\omega_{j}$. The short forms of the variable names have been listed in S1 Table. The likelihood ration test (LRT) has been employed to assess the goodness of fit for the fitted frailty models compared to the standard Cox model (without frailty term) [21]. This test evaluates whether adding a frailty term significantly improves the model fit and follows a chi-square distribution. For the purpose of estimation and test, software R has been used.

## Results

### Univariate and bivariate results

**Fig 1:**
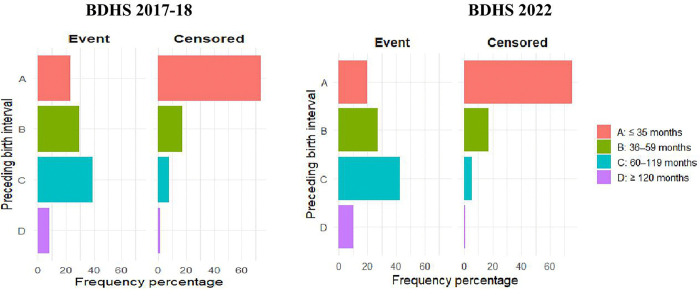
Distribution of preceding birth interval by survival status for urban areas in Bangladesh.

**Fig 2:**
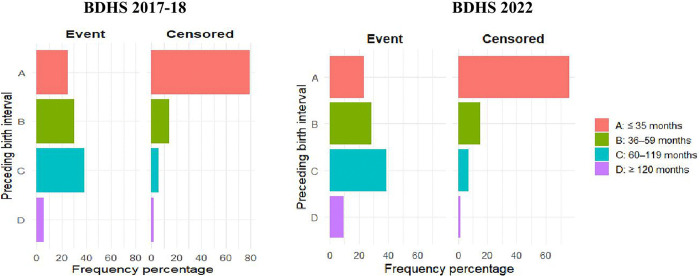
Distribution of preceding birth interval by survival status for rural areas in Bangladesh. Figs 1 and 2 display simple bar diagrams representing percentage frequencies of births intervals. The bars A, B, C, and D stand for the birth intervals of less or equal to 35 months, 36-59 months, 60-119 months, and greater or equal 120 months, respectively. The left and right sided diagrams in Figs 1 and 2 appear for event and censored cases.

The distribution of preceding birth intervals by area for event and non-event has been presented in Figs 1 and 2. In the BDHS 2017−18 survey, among urban women who had a preceding live birth for the index child, a birth interval between 60–119 months possesses the highest frequency (39.0%) and the lowest is observed for the interval of above 119 months (1.7%). However, among censored cases, the majority of the women (73.8%) started their motherhood within three years of their marriages and this frequency of experiencing successful pregnancy decreases as the length of birth interval increases. However, the practice of birth-spacing in rural areas follows a similar trajectory. For BDHS 2022, a similar pattern in birth interval distribution for urban and rural areas has been observed.

The median survival time for birth intervals along with 95% confidence intervals (CI) for urban and rural areas have been provided in Table 1.

**Table 1:** Description of survival time for preceding birth interval by survey years and residence.

Area	BDHS 2017−18				BDHS 2022			
	Percentage		Median survival time (months)	95% confidence interval	Percentage		Median survival time (months)	95% confidence interval
	Event	Censored			Event	Censored		
**Urban**	60.7	39.3	64	(61.871, 66.129)	62.2	37.8	67	(64.580, 69.420)
**Rural**	65.3	34.7	60	(58.454, 61.546)	66.4	33.6	63	(61.361, 64.639)

It has been observed that the median birth intervals for urban and rural areas in the BDHS 2017−18 survey are 64 and 60 months, respectively. However, the 95% confidence intervals for urban and rural areas presented in Table 1 do not overlap and hence provides evidence to justify that median birth interval for rural women is significantly shorter than urban women. In the BDHS 2022 survey, the median birth intervals increase in both urban and rural areas; however, a significantly longer median birth interval continues to be observed among urban women compared with their rural counterparts.

**Fig 3:**
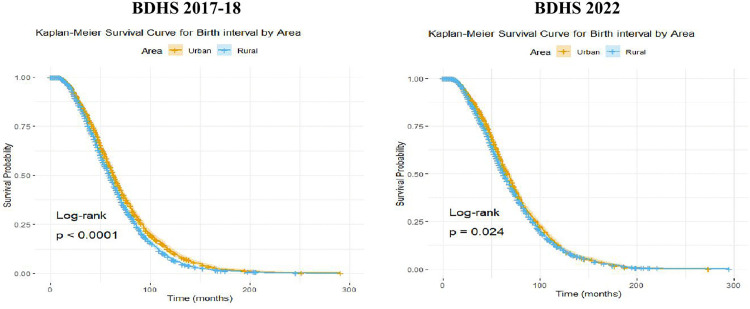
K-M curves of birth interval for urban and rural areas. Fig 3 displays the K-M survival curves of birth interval for urban and rural areas with Log-rank test p values.

Fig 3 shows the Kaplan and Meier (K-M) survival curves of birth interval in both the survey years illustrating the probability of reproductive-aged women remaining without a subsequent birth over time, stratified by urban and rural residence in Bangladesh. In BDHS 2017−18, the curves indicate that, at every time point, rural women have consistently lower survival probabilities compared to their urban counterparts. This suggests that the birth interval is generally shorter among rural women, reflecting more frequent childbirths. The observed difference in birth interval patterns between urban and rural areas is statistically significant, as evidenced by a log-rank test p-value of <0.0001. In the BDHS 2022 survey, although a slight difference in the Kaplan-Meier survival curves between urban and rural areas is visually apparent, the log-rank test confirms that this difference is statistically significant.

**Fig 4:**
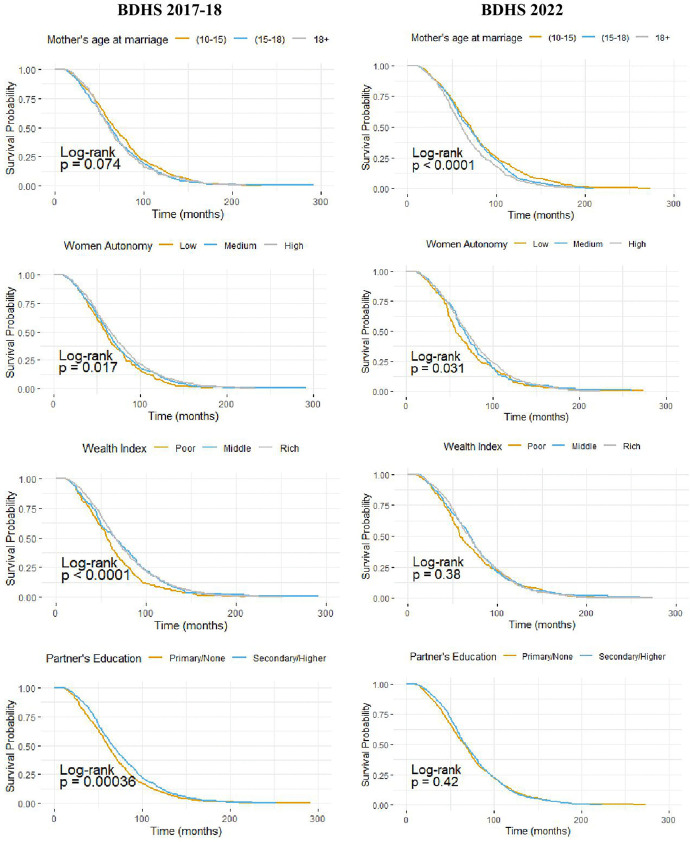
Survival curves of birth interval by some important factors for urban areas of Bangladesh.

**Fig 5:**
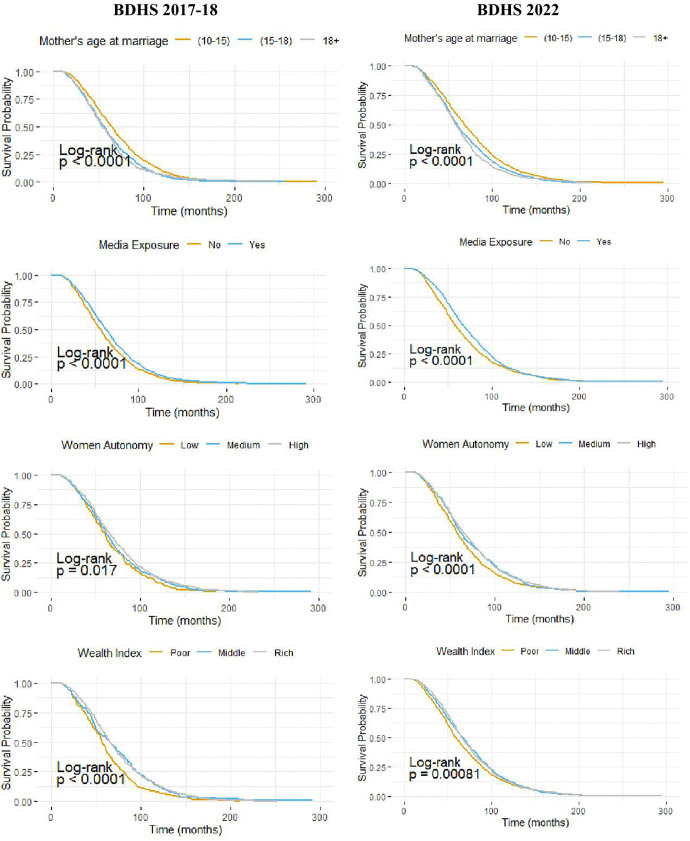
Survival curves of birth interval by some important factors for rural areas of Bangladesh. Figs 4 and 5 display the Kaplan–Meier survival curves for birth intervals across categories of key variables, stratified by survey years for urban and rural areas, along with log-rank test p-values.

Figs 4 and 5 present the Kaplan-Meier survival curves of birth intervals across selected key variables, stratified by survey year for urban and rural areas in Bangladesh, respectively. In urban areas, both surveys consistently indicate significantly shorter birth intervals among women older than 15 years and among those with limited participation in household decision-making. Furthermore, in the BDHS 2017–18 survey, women from lower economic strata and those whose partners have no education or only primary education exhibit significantly more frequent births. However, the BDHS 2022 survey does not show any significant unadjusted association between the wealth index or the partner’s education and birth interval. In rural areas, both survey rounds show that women aged 10–15 years, those with access to media, those with higher levels of participation in household decision-making, and those belonging to affluent households experience significantly longer birth intervals.

Table 2 summarizes the distribution of key background characteristics and their unadjusted associations with birth interval across survey years and residence. In the BDHS 2017−18 survey, early marriage remains prominent in rural areas, where 43% of women married before age 15, compared with 34.1% in urban areas. Media exposure shows a clear urban-rural divide, with 79.9% of urban women reporting exposure versus only 56.9% in rural areas. Women’s autonomy is also notably higher in urban settings, where 53.7% fall in the high-autonomy group, compared with 47.2% in rural areas. A strong socioeconomic gradient is observed: in urban areas, 68.8% of women belong to the richest households, whereas the proportion is only 26% in rural regions. Partner’s secondary or higher education is also more common in urban areas (60.2%) than in rural areas (47.1%).

**Table 2:** Frequency percentage of the selected background characteristics for urban and rural areas along with Log-rank test p-values.

Variables	BDHS 2017−18				BDHS 2022			
	Urban		Rural		Urban		Rural	
	Frequency percentage	p-value	Frequency percentage	p-value	Frequency percentage	p-value	Frequency percentage	p-value
**Mother’s Age at Marriage (Years)**								
(10-15)	34.1	0.074	43.0	<0.001	29.7	<0.001	35.3	<0.001
(15-18)	41.5		43.0		39.6		42.4	
18+	24.4		14.0		30.7		22.3	
**Mother’s Education**								
Primary/none	31.0	0.561	37.4	0.036	26.0	0.194	31.1	0.896
Secondary/Higher	69.0		62.6		74.0		68.9	
**Media Exposure**								
No	20.1	0.001	43.1	<0.001	28.9	0.092	49.1	<0.001
Yes	79.9		56.9		71.1		50.9	
**Women Autonomy**								
Low	18.0	0.017	23.7	0.002	22.7	0.031	25.9	<0.001
Medium	28.3		29.1		11.8		11.2	
High	53.7		47.2		65.5		62.9	
**Exposure to Violence**								
No	85.6	0.325	79.9	0.479	89.1	0.120	87.3	0.739
Yes	14.4		20.1		10.9		12.7	
**Religion**								
Islam	91.4	0.636	90.7	0.394	89.6	0.630	91.8	0.625
Others	8.6		9.3		10.4		8.2	
**Region**								
Dhaka	24.9	<0.001	9.8	<0.001	21.7	<0.001	11.8	<0.001
Chattogram	15.9		16.2		19.9		15.2	
Barisal	9.2		11.0		10.8		11.6	
Khulna	11.2		10.8		10.3		11.9	
Mymensingh	8.3		13.4		8.2		13.7	
Rajshahi	9.2		12.0		10.9		10.3	
Rangpur	9.9		12.5		8.9		13.0	
Sylhet	11.4		14.3		9.3		12.5	
**Wealth Index**								
Poor	18.2	<0.001	53.2	<0.001	18.4	0.379	51.5	<0.001
Middle	13.0		20.8		19.1		20.6	
Rich	68.8		26.0		62.5		27.9	
**Partner’s Education**								
Primary/none	39.8	<0.001	52.9	0.001	37.3	0.423	49.7	0.003
Secondary/Higher	60.2		47.1		62.7		50.3	
**Spousal Age Difference (Years)**								
Mean	7.730		7.89		7.283		7.606	
Stand. deviation	4.648		4.805		7.840		4.915	
Median	7.0		7.0		7.0		7.0	
**Total**	*n* = 2623		*n* = 4761		*n* = 2505		*n* = 4926	

In BDHS 2022, similar patterns persist. Urban women demonstrate substantially higher media exposure (71.1%) than rural women (50.9%). High autonomy is reported by 65.5% of urban respondents compared to 62.9% in rural areas. Wealth disparities remain marked with 62.5% of urban women belong to the richest households, while only 27.9% of rural women fall into this category. Partner’s secondary or higher education continues to be more prevalent in urban settings (62.7%) than rural ones (50.3%). Overall, significant differentials in birth interval are observed across most sociodemographic factors. In both surveys, mother’s age at marriage, women’s autonomy, media exposure, and regional location show strong associations with birth spacing in both urban and rural settings. Wealth index and partner’s education demonstrate significant gradients in BDHS 2017−18, although these patterns largely disappear in the BDHS 2022 urban sample. Rural areas in both survey years consistently show longer birth intervals among women with high autonomy, media exposure, higher wealth status, and partners with secondary or higher education.

### Results from Cox-frailty survival regression model

Table 3 displays the hazard ratios (HR) along with the corresponding 95% confidence intervals obtained from Cox frailty model presenting the relationship among mothers’ socio economic and demographic characteristics to the birth interval by survey year and type of residence.

**Table 3:** Cox-Frailty survival regression model estimates for urban and rural areas in Bangladesh by year of survey.

Variables	BDHS 2017−18						BDHS 2022					
		Urban			Rural			Urban			Rural	
	Coef	HR	95% CI	Coef	HR	95% CI	Coef	HR	95% CI	Coef	HR	95% CI
***Age at Marriage (Years)***												
(10-15)		1			1			1			1	
(15-18)		1.141	1.025, 1.380		1.223	1.130, 1.324		1.096	0.973, 1.235		1.123	1.035, 1.219
18+		1.189	1.025, 1.380		1.332	1.175, 1.509		1.318	1.145, 1.519		1.177	1.057, 1.310
***Mother’s Education***												
Primary/none		1			1			1			1	
Secondary/Higher		1.111	0.985, 1.254		1.067	0.981, 1.161		1.149	1.001, 1.306		1.136	1.044, 1.235
***Media Exposure***												
No		1			1			1			1	
Yes		1.003	0.871, 1.154		0.950	0.876, 1.031		0.966	0.858, 1.088		0.954	0.883, 1.031
***Women Autonomy***												
Low		1			1			1			1	
Medium		0.873	0.743, 1.025		0.895	0.804, 0.996		0.872	0.723, 1.051		0.889	0.780, 1.013
High		0.815	0.703, 0.945		0.903	0.819, 0.997		0.821	0.720, 0.935		0.851	0.780, 0.929
***Exposure to Violence***												
No		1			1			1			1	
Yes		1.017	0.880, 1.174		0.975	0.891, 1.066		0.916	0.780, 1.076		1.013	0.913, 1.124
***Religion***												
Islam		1			1			1			1	
Others		0.853	0.705, 1.033		0.842	0.732, 0.968		0.928	0.780, 1.104		0.896	0.781, 1.032
***Division***												
Dhaka		1			1			1			1	
Chattogram		1.246	1.055, 1.472		1.286	1.100, 1.504		1.329	1.133, 1.559		1.513	1.285, 1.782
Barisal		0.964	0.782, 1.189		1.016	0.857, 1.206		1.048	0.870, 1.264		1.105	0.929, 1.314
Khulna		0.818	0.674, 0.994		0.805	0.678, 0.956		0.946	0.776, 1.152		0.870	0.738, 1.034
Mymensingh		1.131	0.915, 1.399		0.937	0.795, 1.103		1.140	0.929, 1.399		1.180	0.998, 1.394
Rajshahi		0.756	0.615, 0.929		0.710	0.600, 0.839		0.924	0.761, 1.123		0.799	0.669, 0.953
Rangpur		0.933	0.767, 1.136		0.860	0.729, 1.016		0.907	0.738, 1.117		0.932	0.786, 1.106
Sylhet		1.494	1.240, 1.800		1.604	1.364, 1.885		1.496	1.227, 1.825		1.798	1.511, 2.139
***Wealth Index***												
Poor		1			1			1			1	
Middle		0.803	0.667, 0.967		0.841	0.759, 0.931		0.940	0.797, 1.108		0.895	0.810, 0.988
Rich		0.738	0.630, 0.865		0.780	0.700, 0.868		0.890	0.767, 1.033		0.853	0.774, 0.941
***Partner’s Education***												
Primary/none		1			1			1			1	
Secondary/Higher		0.837	0.744, 0.942		0.929	0.851, 1.014		0.920	0.814, 1.041		0.935	0.862, 1.014
***Spousal Age Difference (Years)***	0.052***	1.054	1.024, 1.084	0.021*	1.021	1.002, 1.040	0.015	1.016	0.993, 1.040	0.025**	1.026	1.008, 1.044
***Squared Age Difference***	−0.002**	0.998	0.997, 0.999	−0.001^+^	0.999	0.998, 1.000	−0.001	0.999	0.998, 1.000	− 0.001**	0.999	0.998, 0.999
***Cluster variance***	0.011			0.019			<0.001			0.046		
***LRT statistic***	35.133^**^			114.190^***^			0.001**			259.410***		

*Note*: ^+^p-value < 0.10; *p-value < 0.05; **p-value < 0.01; ***p-value < 0.001.

### Findings from BDHS 2017−18 data

From Table 3, it is clear that the respondents whose age at marriage is between 15–18 years and above 18 years have 1.141 times and 1.189 times the hazard of experiencing birth differentiated to respondents whose age at marriage is between 10–15 years for urban areas. On the other hand, for rural areas these hazards are 1.223 times and 1.332 times, respectively. When the decision-making autonomy is medium and high the hazard of birth outcome for urban mothers are respectively 12.7% and 18.5% lower while these hazard are 10.5% and 9.7% lower for the rural mothers compared to those who have less access to decision-making autonomy. An important finding in this research is that there is a significant impact of religion on the birth interval in the rural areas, in contrast religion has not been found to be a significant determinant of the birth interval in urban areas. Non-Muslim mothers have 15.8% lower hazard of childbirth compared to the Muslim mothers. In the case of the region, the respondents who belong to Chattogram and Sylhet have respectively 1.246 and 1.494 times the hazard of experiencing birth event compared to the respondents who belong to Dhaka in urban areas, whereas these chances are 1.286 times and 1.604 times for rural areas. However, the respondents from Khulna and Rajshahi have significantly longer birth intervals compared to the respondents from Dhaka in both urban and rural areas. The respondents from middle-class and rich families have longer birth intervals compared to the respondents from poor families in both urban and rural areas. The respondents who belong to middle-class families and rich families have respectively 19.7% and 26.2% lower hazard of birth occurrence compared to those who belong to a low-income family in the urban areas, whereas these hazards are 15.9% and 22% lower in the rural areas. More specifically, respondents whose partners are secondary or higher educated have 16.3% lower hazard of experiencing a birth compared to respondents whose husbands are uneducated or have at most primary education level in the urban areas that is they have the longer birth interval. Though it is observed that in rural areas partners’ education have not shown any significant effect on the birth interval.

Mother’s education, media exposure and violence towards women do not show any significant impact on birth intervals in urban as well as in rural areas of Bangladesh. In the case of spousal age difference, both the linear and squared effects have a significant impact on birth interval. It has been observed that for each additional increase of the age difference the hazard increases by 5.4% in urban areas and 2.1% in rural areas. As the coefficient for squared age difference is found negative (−0.002, p-value<0.01), it is implied that initially with the increase of age difference of husbands and wives, the hazard of giving a birth increases but after a certain point of age difference, this hazard starts to decrease. The estimated age differences at which the hazard of experiencing a birth is maximum are 13 years and 10.5 years for urban and rural areas, respectively. Moreover, the variances of the random effects are 0.011 in urban areas and 0.019 in rural areas. According to the likelihood ratio test p-values, the preferred Cox-frailty model provides a good fit compared to the standard Cox model (without a frailty term) for both urban and rural Bangladesh.

### Findings from BDHS 2022 data

There are noticeable discrepancies in the results derived from the BDHS 2022 data. In rural areas, respondents who married at ages 15–18 years and above 18 years have 1.123 and 1.177 times the hazard of giving birth compared to those who married at ages 10–15 years. In urban areas, however, the 15–18 years age at marriage category is no longer a significant predictor of birth interval. Unlike the BDHS 2017−18 data, the variable ‘mother’s education’ becomes significant. Respondents with secondary or higher education have 14.9% and 13.6% higher hazard of experiencing a birth compared to respondents who are uneducated or have at most primary education level, respectively, in urban and rural areas. For urban mothers, having high decision-making autonomy is associated with a 17.9% lower hazard of birth compared to those with low autonomy. In rural areas, however, medium autonomy is not a significant predictor of birth interval, which was previously significant in the 2017−18 data. Also, religion does not significantly influence the birth interval in either area. In the case of the region, the respondents who belong to Chattogram and Sylhet have respectively 1.329 and 1.496 times the hazard of experiencing birth event compared to the respondents who belong to Dhaka in urban areas, whereas these chances are 1.513 times and 1.798 times for rural areas. However, the respondents from rural areas of Rajshahi have significantly longer birth intervals compared to the respondents from Dhaka. Individuals from middle and high-income households exhibit 10.5% and 14.7% lower birth hazard rates, respectively, compared with their counterparts in low-income families in the rural areas. In urban areas, the variable ‘Wealth Index’ now appears insignificant, though it was significant earlier. Partners’ education has not shown any significant effect on the birth interval in both areas. For rural areas, the hazard of experiencing a birth increases by 2.5% for each additional year of age difference, but given the negative squared age difference coefficient (- 0.001, p-value< 0.01), which indicates a downturn after a threshold, this hazard is estimated to be highest at a 12.5 year age difference. Discrepancies have also been observed for the age difference in urban areas; neither the linear nor the quadratic age difference effects are statistically significant. Moreover, the variances of the random effects are < 0.001 in urban areas and 0.046 in rural areas. According to the likelihood ratio test p-values, the preferred Cox-frailty model provides a good fit compared to the standard Cox model (without a frailty term) for both urban and rural areas in Bangladesh.

## Discussion

This study is dedicated to address the variation of impact of several socio-economic and demographic variables on preceding birth intervals of mothers in rural and urban areas of Bangladesh. The median birth intervals in rural and urban mothers are found to be 60 months and 64 months, respectively in BDHS 2017−18 data. The difference observed can be attributed to the existing socio- cultural gap in the two studied areas. However, most of the covariates considered are found to have similar impact on birth interval in BDHS 2017−18 data, discrepancy is only observed for the variables religion and partner’s education in rural and urban Bangladesh. It is found that, Muslim mothers living in rural areas tend to have shorter birth intervals compared to the mothers from other religions. This result is similar to the findings of another study conducted in rural Bangladesh [22]. Young Muslim couples with high religious beliefs in Iran desire to have many children [23]. Similarly, Muslim women living in the Northern part of Nigeria generally want to have big families, as a consequence make less use of contraceptives [24]. In Bangladesh it is observed that Muslim mothers are more likely to have unmet need for family planning compared to non-Muslim mothers [25,26]. Therefore, strong religious beliefs among rural mothers might bring down their use of contraceptives and hence make the birth interval shorter for them. However, religion showed no significant impact on birth intervals among urban mothers in BDHS 2017–18 data and remained insignificant across both rural and urban areas in BDHS 2022 data.

Previous research [27] indicates that respondents who are married to partners who have secondary or higher education have lower likelihood of being a mother compared to those who are married to partners who have primary education or no education. Educated partners may have a better understanding of the importance of proper birth spacing, which may put an effort to make use of modern contraception to widen out the birth intervals. It is also revealed in an earlier study that partners with higher education have significantly lower unmet need for family planning compared to those who have below higher education [25]. Other researches indicate that fathers with advanced education are more inclined to support their partners in making informed choices about reproductive and healthcare matters [28,29]. However, this study unveils a distinctive trend, urban mothers whose partners have secondary or higher education experience longer birth intervals compared to the mothers whose partners have primary or no education, on the other hand in the case of rural mothers, partner’s education remains insignificant to their birth intervals. This disparity in the impact of partner’s education between urban and rural areas suggests a complex interplay of contextual factors. Partner’s education may remain insignificant to mothers’ birth intervals due to the factors such as social barriers, poor health care facilities, distance to health care center and cultural milieu in the rural areas. However, the BDHS 2022 data indicate that, partner’s education has no significant effect in both areas.

A significant association between mother’s age at marriage and birth interval has been observed for both the urban and rural areas in BDHS 2017−18 data. We have found that women married at older age tend to have shorter birth intervals than those married at younger age. This is because, couples are more concerned nowadays regarding infertility and incidence of congenital diseases at increased maternal age and therefore willing to have children early after marriage. However, this finding is unlike that reported by some past studies conducted in Bangladesh [1,30] but similar with the research work carried out in neighboring country Nepal [31]. Similar results for mother’s age at marriage have been observed in BDHS 2022 data.

In several studies it was reported that women with no education tend to have shorter birth intervals compared to their counterparts of educated women [16,31]. However, in this study, mother’s education has not shown any significant impact on the birth intervals in both urban and rural areas in BDHS 2017−18. Surprisingly, the 2022 data show that women with secondary or higher education tend to have shorter birth intervals than those with primary or no education. This could be due to advanced age at marriage among the educated mothers, prompting them to accelerate childbearing relative to others.

As expected, it is observed that women with medium and high decision-making autonomy have shorter birth intervals compared to those with low autonomy in both rural and urban areas in both the considered data sets. This result corroborates with the findings of a study conducted in Cebu, Philippines [32]. Another study in Oman has been explored that more empowered mothers are more probable to make use of contraceptives [33]. Women autonomy for daily household purchases and spousal communication is found to be significantly related to their fertility preferences and modern contraception use in Eritrea [34]. Therefore, the use of contraceptives may impede recurrent childbirth of empowered mothers and facilitate longer birth spacing in Bangladesh. It is also observed that women from middle class and rich families in BDHS 2017−18 data tend to have significantly longer birth intervals compared to the poor families, in both urban and rural areas, which is consistent with the previous work in Bangladesh [1]. Reasons behind this could be, women from the wealthier families have greater access to health knowledge, family planning and maternal health care services. In 2022 data wealth index is only found significant in rural area.

In this research spousal age difference showed significant impact on birth interval. It is observed that, birth interval decreases as the age difference increases, though the birth interval again increases after certain points of age differences in urban and rural areas. It can be explained with the fact that younger women might be dominated by their husbands in some cases which brings down independence of their choice of pregnancy. However, partners who are much older than their wives might develop fertility related complications and thereby amplify the length of birth interval. Similar findings have been observed in earlier research in Bangladesh by [35], where it is reported that the waiting time to first birth is lower for the respondents whose spousal age difference is higher. However, in a past study it is concluded that spousal age difference does not have any significant impact on birth interval in Bangladesh [36]. In 2022 data spousal age difference is only found significant in rural area.

## Conclusion

The current study has demonstrated the disparities in the length of birth intervals between the rural and urban areas of Bangladesh and studied the factors associated with birth intervals inboth settings. In BDHS 2017−18 data, the median birth interval is found to be 60 months for rural mothers and 64 months for urban mothers, and in both areas, the contributing factors that are significantly associated with birth intervals are almost the same which are age at marriage, women decision making autonomy, wealth index, division and spousal age difference. Disagreement is only observed for the variables partner’s education and religion between the urban and rural areas. According to BDHS 2022 data, the median birth interval is 63 months for rural mothers and 67 months for urban mothers, indicating that birth spacing increases over time in both areas. The common significant factors of birth intervals in rural and urban areas are age at marriage, women decision making autonomy, and division. In contrast, the effect of wealth index and spousal age difference differ by area. Moreover, mother’s education emerges as significant even though it was insignificant in BDHS 2017−18 data, and interestingly, more educated mothers are having children more frequently, highlighting the need to investigate the reason behind this pattern. From the findings, it is recommended that partners in urban areas should be stimulated to attain higher education and Muslim mothers in rural areas should be enlightened about the importance of adopting modern contraceptives geared towards maintaining optimum birth spacing. Also, policymakers should intensify the existing facilities for raising public knowledge of optimum birth intervals to general people especially to those who are economically less stable residing in rural areas to hinder deaths and illness of the mother and newborn. Besides, in order to improve pregnancy related outcomes, policy and programs should focus on ensuring women autonomy. The limitation of this research is that, the information regarding the birth interval and the covariates included in, was gathered through recalling previous events. As a result, the conclusions of this study do not portray the current situation. Besides the data collected are cross sectional, therefore involve some missing information. Further research can be conducted considering cluster level covariates in the model.

## Supporting information

S1 Table Measurements of the outcome variable and other exposure variables used in the study. (DOCX)
