# Supplementary material for: A cluster level study for the identification of the disparities in birth intervals between rural and urban areas of Bangladesh
Source: PLoS One. 2026 Feb 5;21(2):e0342304. doi: 10.1371/journal.pone.0342304 (PMC12875462; doi:10.1371/journal.pone.0342304)
Supplement: S1 Table — (DOCX) [file pone.0342304.s001.docx]

**S1 Table. Measurements of the outcome variable and other exposure variables used in the study**

| **Variables** | **Definition and Measurement** |
| --- | --- |
| **Outcome Variable** |  |
| Preceding Birth Interval | The outcome variable in this study is preceding birth interval which has been considered as time-to-event variable. In this study, a failure time for event is measured as the preceding birth interval for the index child. If the index child is the first birth of his/her mother, it will be considered as a censored case. In this case, a censoring time (time for non-event) is evaluated as the time between marriage and first birth. |
| **Covariates** |  |
| Mother’s Age at Marriage (MAM) | The categories for Mother’s age at marriage (in years) have been created as 10-15: mothers aged 10 to 15 years, 15-18: mothers older than 15 but up to 18 years, , and 18+: mothers older than 18 years. |
| Mother’s Education (ME) | Mother’s education has been re-categorized as Primary/none and Secondary/Higher. Education level primary and no education are merged and named as Primary/none, whereas mothers who have secondary or higher education level are placed into the Secondary/Higher group. |
| Media Exposure (MEX) | Women who have access to Radio/ Television/ newspaper have been categorized as Yes, and No, if they do not have such access. |
| Women Autonomy (WA) | Women autonomy is created using four variables regarding their involvement in decision-making related to households and health issues available in BDHS, 2017-18 survey. At first these four variables: person who decides on (i) issues related to her own health care, (ii) large household purchases, (iii) how to spend her partner’s earning, and (iv) visits to relatives, have been re-categorized as binary variables taking values 1, if women are involved, and 0, otherwise. Finally, a sum of these binary variables is evaluated resulting in the values 0, 1, 2, 3, or 4. Therefore, based on the scores, the autonomy power has been categorized as Low (0 or 1), Medium (2), and High (3 or 4). |
| Exposure to Violence (EV) | Women who get beaten by their partners for any of these five reasons: if they burn food, refuse to have sex, argue with their partners, go outside without informing their husbands, and show a lack of care for their children, have been categorized as Yes, and No, otherwise. |
| Religion (REL) | In the present study, the variable Religion has been re-categorized into two groups: Islam and Others (Hinduism, Buddhism, and Christianity). |
| Region (REG) | The eight divisions of Bangladesh have been considered as the eight categories of the variable Region. |
| Wealth Index (WI) | The three categories: Poor, Middle, and Rich have been re-formed from actual variables that existed in BDHS, 2017-18 survey. The categories poorest and poorer are combined resulting in the category Poor, whereas the Rich group is created by merging Richer and Richest groups. However, the Middle group remains unchanged. |
| Partner’s Education (PE) | Likewise Mother’s education, Partner’s education has been categorized as Primary/None and Secondary/Higher. |
| Spousal Age Difference (Years) (SAD) | This is a continuous variable measured by subtracting women’s age from their partners’. |
